# Surgeons’ perspective on the use of carbon fibre plates for extremity fracture fixation

**DOI:** 10.1007/s00590-024-04131-1

**Published:** 2024-11-25

**Authors:** F. Shiers-Gelalis, V. Giannoudis, P. Rodham, P. V. Giannoudis

**Affiliations:** 1 Health Education Yorkshire and Humber, St James’ University Hospital, Leeds, UK; 2 Academic Department of Trauma and Orthopaedics, School of Medicine, University of Leeds, Leeds, UK; 3 Leeds General Infirmary, Clarendon Wing, Level A, Clarendon Way, Leeds, LS1 3EX UK

**Keywords:** Carbon fibre, Implant, PEEK, Fracture fixation, Surgeon perspective

## Abstract

**Introduction/purpose:**

Carbon fibre plating (CFR-PEEK) became available to orthopaedic surgeons in 1998 as a competitor to the traditional metal implants. Despite this, the use of such implants has been limited globally. The aim of this study was therefore to explore the barriers to more widespread use of CFR-PEEK, specifically by examining orthopaedic surgeons’ perceptions and opinions of its use through a cross-sectional survey.

**Methods:**

An online questionnaire with basic information attached regarding similarities and differences between CFR-PEEK and metal implants was sent out internationally, with 106 responses gained from 26 countries. Specific questions were asked to ascertain orthopaedic surgeons’ current knowledge of advantages and disadvantages of CFR-PEEK, the barriers they perceive to its more widespread use, and own personal preferences. Free-text responses were analysed and the results discussed.

**Results:**

A minority of orthopaedic surgeons surveyed would choose CFR-PEEK over traditional metal implants (10.38%). The most common disadvantage of CFR-PEEK reported was increased cost, with 46.23% respondents identifying this. Concerns regarding structural integrity of the implant were second most commonly perceived disadvantage, with 34% of surgeons citing one or more of ‘stiffness/breakage/durability/contourability’ as a disadvantage. A small number of surgeons (3.8%) listed unfamiliarity as a potential disadvantage to the use of carbon fibre plates. The main barrier identified to their use was poor knowledge (education) in relation to the properties and existing evidence of their performance.

**Conclusions:**

More work is needed to make CFR-PEEK more acceptable to surgeons including examination of perceived increased cost and increasing education of these implants. Further high-level evidence confirming carbon fibre non-inferiority may increase the usage of CFR-PEEK for extremity fracture fixation in the future.

**Supplementary Information:**

The online version contains supplementary material available at 10.1007/s00590-024-04131-1.

## Introduction

Carbon fibre is a well-established material that been utilised for many years due to its innumerable physical and chemical properties. With its application spanning from initial commercial use in lightbulbs in 1879, to the aerospace industry and its use in the fabrication of rocket nozzles in the 1960s [1], carbon fibre’s relatively inert properties combined with its tensile strength make it an important and valuable commodity. More recently, carbon fibre and its application has evolved to the field of medicine and has been demonstrated to have a plethora of uses within trauma and orthopaedics, from joint implants and spinal cages, to fracture fixation [2]. Noteworthy, since 1998, carbon fibre -reinforced polyetheretherketone (CFR-PEEK) plates were accepted for commercial use as a implant biomaterial for the management of extremity fractures [3].

Their relevant biomechanical properties and advantages can be briefly summarised into: elastic modular properties similar to bone, radiolucency allowing for more accurate fracture reduction and monitoring of healing, reduction of MRI artefact, no recorded incidence of metal allergy and absence of cold-welding at plate/screw interface [4]. However, CFR-PEEK plates have also some disadvantages which are of note, including initial increase in cost to produce, an inability to contour the implants intraoperatively, and although radiolucency can be advantageous with regard to achieving more accurate fracture reduction, by definition this can in turn makes visualising the implant more difficult.

There have been a number of small studies published which indicate that CFR-PEEK plates are at least equivalent and in some cases superior to the standard metal implants [5–7]. However, there is relative paucity of high-level evidence literature such as systematic reviews and randomised control trials demonstrating CFR-PEEK plating being equivalent (or superior) to traditional methods of fracture fixation. Not withstanding, the systematic reviews which have been published so far indicate that CFR-PEEK plates are not inferior to metal implants—with one systematic review by Chuan Silvia Li et al. expressing strong support for the use of CFR-PEEK materials[8] and another by Chloros et al. reporting CFR-PEEK implants as a valid alternative to conventional plating [4].

Recently, work has been undertaken to gain an understanding of patient views and acceptability of undergoing fracture fixation with carbon fibre plates [9], in an effort to increase patient and public involvement prior to the commencement of any large randomised control trial. This study found that when supplied with clinical evidence for both CFR-PEEK plates and metal implants, a significant majority of patients would opt to have their fracture fixed with carbon fibre and would be amenable to being involved in a randomised control trial to this effect.

When examining barriers to the more commonplace use of CFR-PEEK plates, it is also important to consider the perceptions of those most likely to utilise them, specifically the orthopaedic surgeons.

The aim of this study is therefore to explore the barriers to more widespread use of carbon fibre plating, specifically by examining orthopaedic surgeons’ perceptions and opinions of its use and understanding reasons for choosing traditional metal implants over the newer carbon fibre implants, or vice versa.

## Materials and methods

An online cross-sectional survey of orthopaedic surgeons was carried out. The inclusion criteria to take part in the survey were any orthopaedic surgeon with 1 year of experience or more. A link to the survey was sent out internationally, and responses from 26 different countries were received. These results were collected from 02/11/2022 to 26/03/2024.

The survey was comprised of 8 distinct questions, some of which were free text (Appendix 1), and respondents were provided with the same basic information prior to completing the questionnaire (see Appendix 2). The questions aimed to ascertain surgeons’ knowledge of the existing differences, advantages and disadvantages of carbon fibre plating, as well as their own personal preference. The information provided consisted of summarising the key differences between carbon fibre and metal implants, with reference to a number of factors including composition of implants, radiological findings and relative cost of the implants.

Due to the qualitative nature of the data, specific statistical analysis is limited. However, data regarding commonly occurring beliefs and preferences have been gathered, along with some basic demographics of the respondents. The responses to the questionnaire were tabulated, and the numbers of respondents who opted for CFR-PEEK versus metal implants, along with their rationale analysed.

## Results

**Fig. 1:**
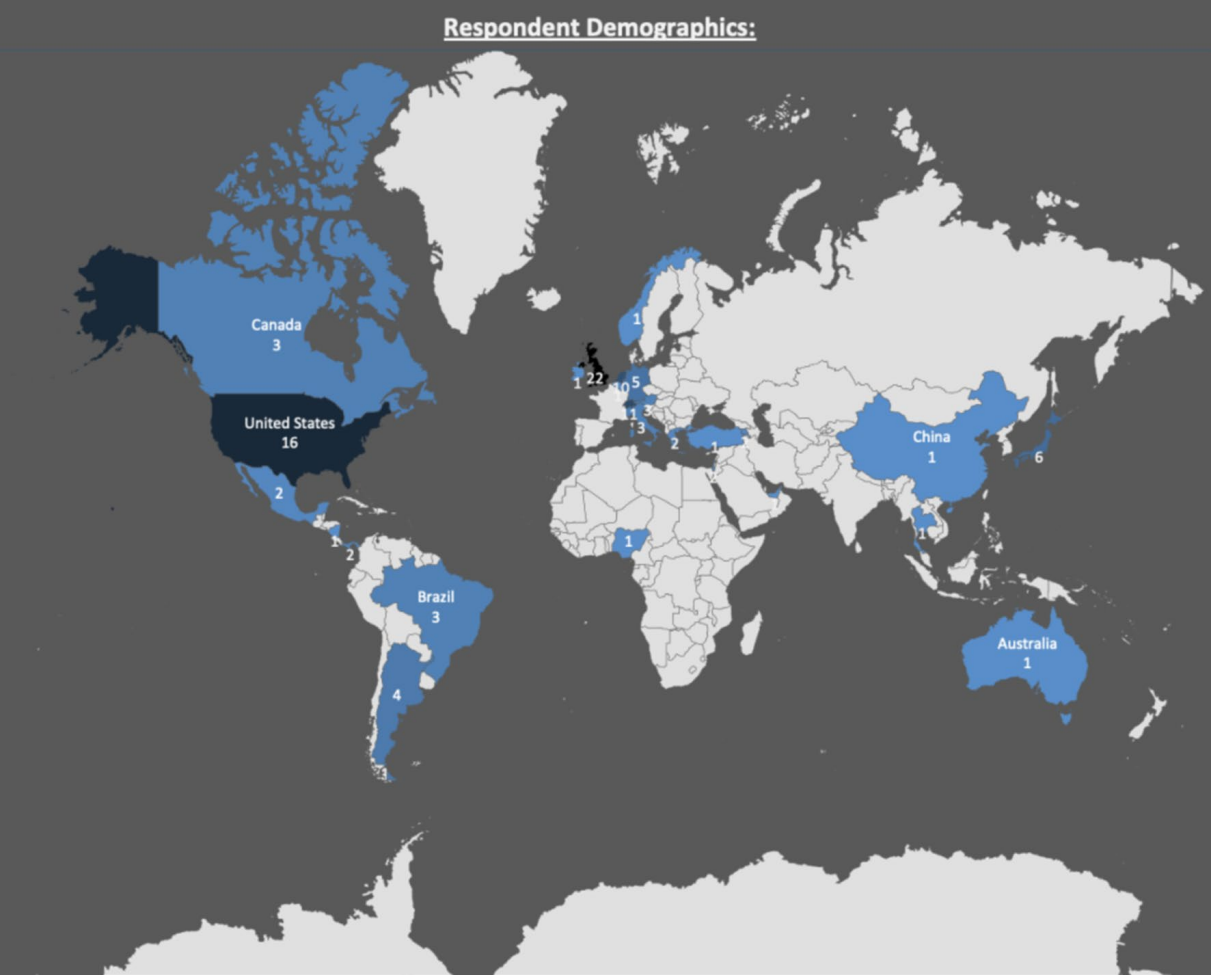
World map showing survey responses from orthopaedic surgeons globally

There were 106 respondents to the survey, practicing in 26 different countries (Fig. 1). The majority of respondents were from the UK (22/106), and of these 27.3% (6/22) would opt to use CFR-PEEK or be equally likely to use it as metal implants.

**Fig. 2:**
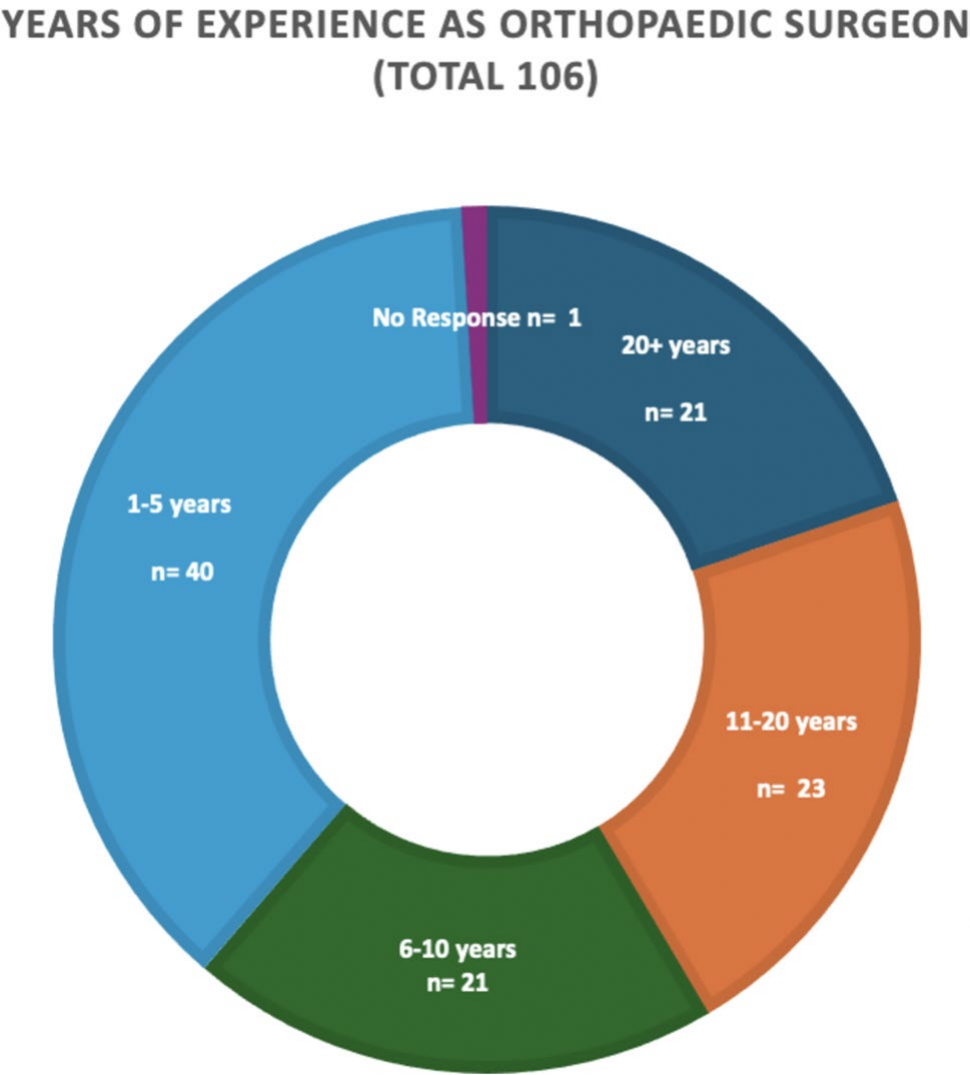
Pie chart showing the distribution of orthopaedic surgeon experience to survey response

Of those surveyed, the most common level of experience were those surgeons who had 1–5 years of experience at registrar level or above: 40/106 (37.7%), and metal implants were the most popular choice of device at every experience level (Fig. 2).

There were 8 distinct questions asked of respondents, with two of these being solely free text in nature. The first asked about the possible disadvantages of carbon fibre plating versus metal implants, and the second asked for the possible advantages of carbon fibre plating. Broadly the responses could be characterised as pertaining primarily to concerns regarding cost, comments on biomechanics (with words such as ‘countourability’, ‘rigidity’, ‘strength’ and ‘flexibility’ being used), radiolucency, or commonly ‘unsure’ or ‘I don’t know’ was written.

Out of the responses, 70/106 (66.1%) reported being familiar or partially familiar with the differences between metal and CFR-PEEK. Of those, the highest percentage respondents (26/70, 37.1%) had 1–5 years of experience as an orthopaedic and trauma surgeon.

**Fig. 3:**
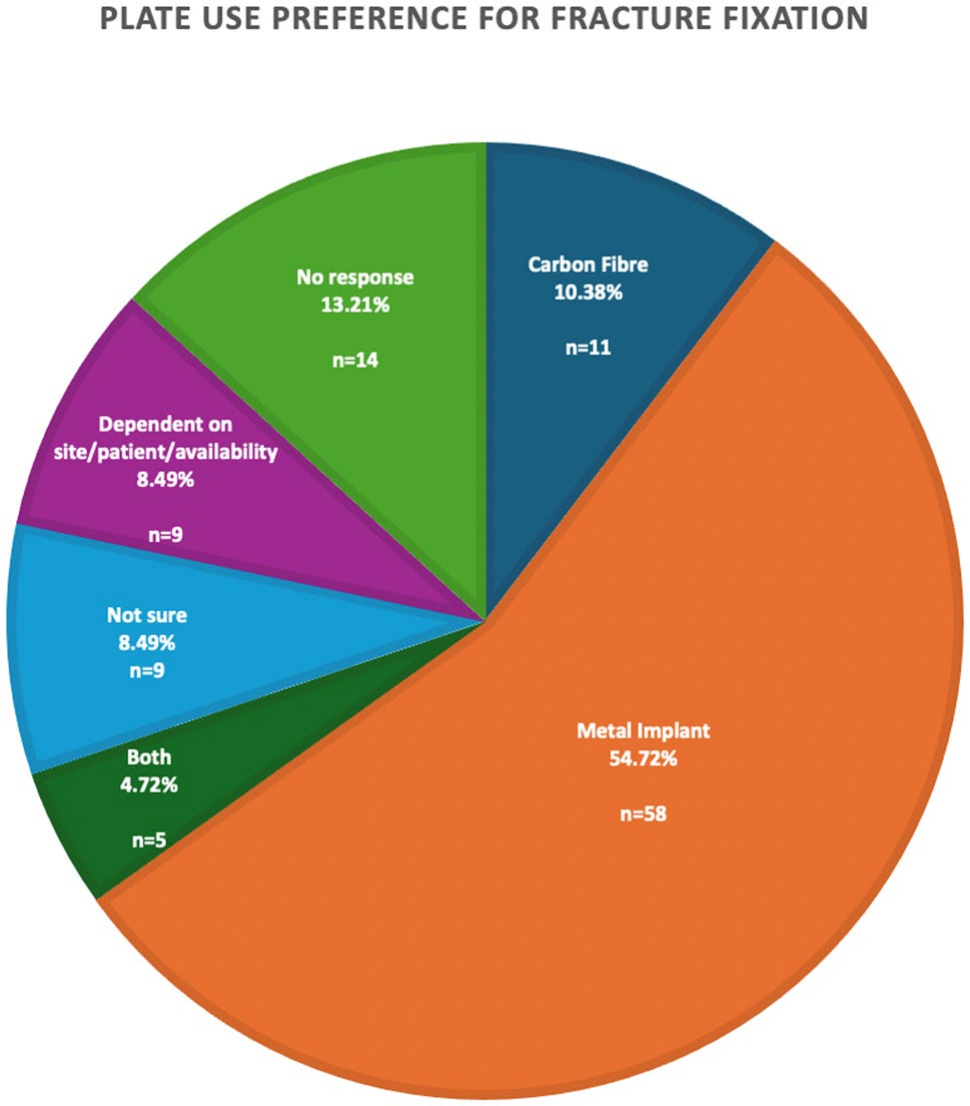
Pie chart showing surgeon preference for fracture fixation (CPPK vs Metal implants)

When given the choice of which implant to use for fracture fixation (Fig. 3), the majority of respondents chose to use a metal implant (58/106, 54.7%). 10.38% would choose CFR-PEEK over the traditional metal implant, and 4.7% of those surveyed would choose both equally. 9/106 surgeons that responded to the survey stated their choice would depend on the fracture site, different patient factors and the availability of the implant. A further 9/106 wrote that they were not sure which to choose. Of note, a significant proportion of those surveyed, 14/106 (13.2%) did not provide any response to this question.

In terms of the numbers of respondents who had actually used CFR-PEEK previously, 14/106 (13.2%) reported having used them—with only one of these respondents opting to use carbon fibre plating if given the choice. Interestingly, 9/14 (64.2%) who had used CFR-PEEK before, but who opted to use metal implants if given the choice, listed disadvantages to them as: cost, fibre debris, radiolucency, concerns regarding tensile strength, and lack of experience in their use.

**Fig. 4:**
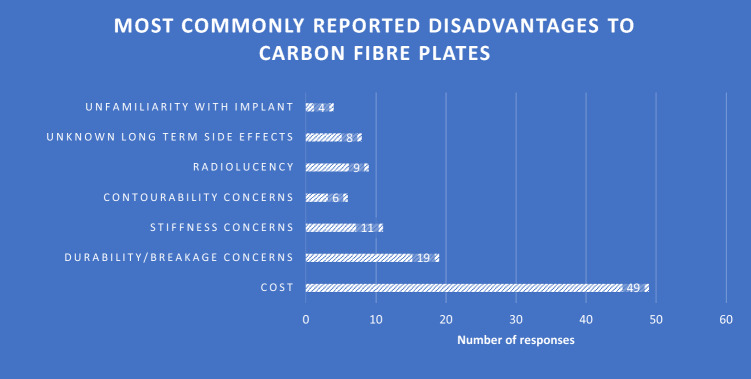
Survey summary of disadvantages relating to CFR-PEEK Plates for fracture fixation

Globally the most commonly reported disadvantage to using CFR-PEEK, in those who were both familiar and unfamiliar with them was the perception of increased cost, with 49/106 respondents (46.2%) writing ‘cost’ or ‘expense’ (Fig. 4). Concerns regarding structural integrity of the implant was second most commonly perceived disadvantage, with 34% of surgeons citing one or more of ‘stiffness/breakage/durability/contourability’ as a disadvantage. 3.8% of surgeons listed unfamiliarity as a potential disadvantage to the use of carbon fibre plates.

Advantages of using CFR-PEEK were most commonly listed as radiolucency, allowing for more exact fracture reduction, with 50.9% of surgeons mentioning this. The second most commonly recorded advantage, listed by 34.0% of those surveyed, was the perceived useful biomechanical properties of carbon fibre—specifically its strength, flexibility and stability. Other popular advantages to carbon fibre plating were listed as it being lightweight, biocompatibility, and a reduction in imaging artefact with both MRI and CT.

**Fig. 5:**
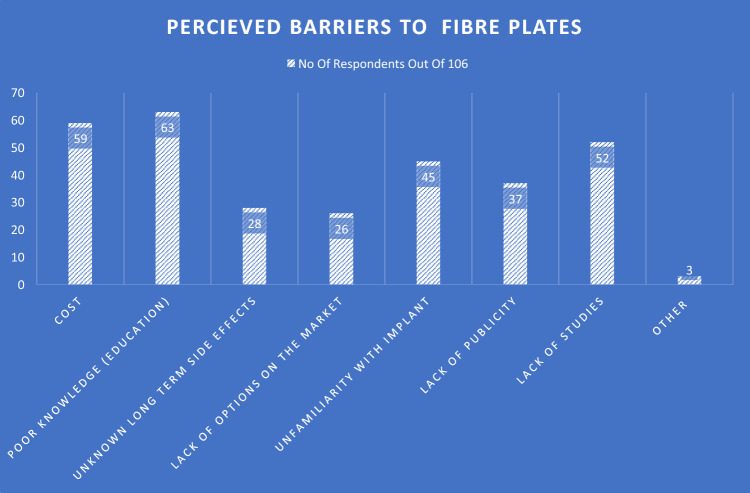
Survey summary assessing themes regarding perceived

When analysing responses to the question regarding what surgeons perceived the barriers to the usage of CFR-PEEK (Fig. 5), the most commonly reported factor was poor knowledge (education) with 63/106 (59.4%) respondents identifying this as one of the factors. Second most common perceived barrier was cost, with 59/106 (55.7%) reporting cost as a barrier to carbon fibre plate use. Unfamiliarity with the CFR-PEEK was also a commonly chosen barrier to their use, with 45.5% listing this as one. There were 3 respondents who reported potential barriers that were not listed, which included lack of availability within NHS, and previous composite implant failure.

Pearson Chi-squared tests were used to examine the relationship between years of experience and whether respondents would choose CFR-PEEK plating, metal implants, or both, but there was no statistical significance (*p* = 0.728). There was also no statistical significance when correlating familiarity with use of CFR-PEEK plating with their respective choice of implant (*p* = 0.756).

## Discussion

The results of this survey indicate that there is a clear lack of surgical preference for the use of CFR-PEEK in the fixation of fractures. The reasons for this may lie in both the perception of increased cost of the implant, and a lack of knowledge of the implant itself. This is demonstrated by the significant majority of surgeons opting to use metal implants if given the choice, the cost being listed as the most common disadvantage to their use, and the selection of ‘poor knowledge (education)’ as the most commonly perceived barrier.

Despite only 13% of respondents ever having used the plate in the past, it is interesting that only 4% listed unfamiliarity with the implant as a disadvantage to carbon fibre plating. However, when asked in the questionnaire to select the barriers to their usage, 45.5% chose unfamiliarity with the implant as one of them. This demonstrates that unfamiliarity with using carbon fibre plates is not seen as disadvantageous to the individual surgeon but is perceived as a barrier to their use more globally. Radiolucency was also listed by 9/106 respondents as a disadvantage. However, implants can now be made with tantalum markers to confirm plate position radiographically [10].

The majority of respondents to the survey were surgeons with 1–5 years of experience, and CFR-PEEK was more familiar to this group than at any other experience level. This is interesting as the use of CFR-PEEK plating is an emerging and evolving landscape, and this level of awareness in this group rather than in those with more experience is surprising.

Analysis of responses regarding advantages demonstrates that the respondents are more aware of radiolucency as an advantage in the use of carbon fibre plating, allowing for more accurate fracture reduction. However, it is also worth noting that its radiolucency was also listed as a perceived disadvantage to their use. A number of other advantages such as biomechanics and biocompatibility, along with reduced artefact in MRI, were mentioned, indicating a significant level of awareness of the advantages of CFR-PEEK plating over metallic implants, despite poor knowledge being selected as a barrier to their use most commonly.

Another notable finding when analysing these results is that of the 14/106 surgeons that reported having used carbon fibre plates in the past, only one of those opted to use them again if given the choice. In this group, the disadvantages of CFR-PEEK plates listed included cost, radiolucency, stiffness, and the perception of in increased propensity to break and produce fibre debris. This is an interesting finding, as the literature developed so far does not identify these as commonplace in the use of CFR-PEEK.

When examining the free-text responses further, the biomechanics of carbon fibre plating are often mentioned in both advantages and disadvantages, with words sight as ‘lighter’/’light weight’, ‘countourability’, ‘flexibility’ and ‘stiffness’ being listed as advantageous properties. Thematically, when looking at free-text answers pertaining to the possible disadvantages of CFR-PEEK plating, concerns surrounding the material’s density and durability were common, with words such as ‘less dense’, ‘breakage’ and ‘shattering’ being used. This is interesting as it betrays a feeling amongst surgeons that CFR-PEEK is not felt to be as durable as proven in the literature. However, the most common word by far used when discussing the possible disadvantages of carbon fibre plating was ‘cost’.

To our knowledge, this is the first study assessing surgeons’ experiences in relation to use of CFR-PEEK implants. It has highlighted potentials barriers to use and the need for implant manufacturers to implement clear education programmes to surgeons early in their career pathway e.g. surgical residency to allow them to become more familiar with these implants.

Based on the nature of the survey, there may be some potential shortcomings. Firstly, there is underrepresentation of certain continents (Oceania, Asia and Africa) which may have altered our findings. Secondly, it is difficult to exclude that participants had no knowledge at all of CFR-PEEK and their initial exposure to them may have come from the information leaflets provided initially at the start of the survey. Moreover, the fact that only 13% of respondents had actually used the plates in the past, results in a potential bias of those surveyed, as the overwhelming majority had no prior experience with the CFR-PEEK plates.

## Conclusion

In conclusion, this study aimed to evaluate orthopaedic surgeons’ perceptions of carbon fibre plating, its perceived advantages and disadvantages in comparison to traditional metal implants, and the barriers to their usage more widely. The results of this survey indicate that there is an overwhelming majority of surgeons who would prefer to utilise metal implants over carbon fibre. In order to increase the utilisation of carbon fibre plating for the fixation of extremity fractures, more work needs to be done in order to make them more acceptable to surgeons through detailed examination of their perceived increased cost, lack of knowledge and concerns regarding their structural integrity. The synthesis of more high-level evidence confirming CFR-PEEK non-inferiority could result in increased usage within the surgical community.

## Supplementary Information

Below is the link to the electronic supplementary material.

Supplementary file1 (DOCX 16 KB)
