# Supplementary material for: Surgeons’ perspective on the use of carbon fibre plates for extremity fracture fixation
Source: Eur J Orthop Surg Traumatol. 2024 Nov 25;35(1):26. doi: 10.1007/s00590-024-04131-1 (PMC11588834; doi:10.1007/s00590-024-04131-1)
Supplement: Supplementary file 1 — Supplementary file1 (DOCX 16 KB) [file 590_2024_4131_MOESM1_ESM.docx]

**Surgeons’ perspective on the use of carbon fibre plates for extremity fracture fixation.**

**Appendix 1**- Survey sent to orthopaedic surgeons

1. How many years have you been in practice as an Orthopaedic and Trauma Surgeon?
2. Which country do you practice in?
3. Were you familiar with the existing differences between metal and carbon fibre plates? (please select)

- No, and I have not used the plates
- No, but I have used the plates
- Partially, and I have used the plates
- Partially, but I have not used the plates
- Yes, and I have used the plates
- Yes, but I have not used the plates

**What do you think the key advantages of a carbon plate versus a conventional one might be?**

1. What do you think the key advantages of a carbon plate versus a conventional one might be?
2. What do you think the key disadvantages of a carbon plate versus a conventional one might be?
3. If you have the choice, to use either plate for a fracture fixation, which one would you prefer to use and why? (Please write below)
4. Would you be happy to take part in a randomized control trial (research study) in the future that would compare metal versus carbon plate implants?
5. Although carbon fibre plates have been in the market for over a decade what do think have been the barriers to their extensive use in the clinical setting? Tick all that apply. (Please write any further reasons that are not covered below)
6. Poor of knowledge - education
7. Lack of studies
8. Lack of publicity
9. Cost of treatment
10. Unknown long term site effects if any
11. Unfamiliarity with their use
12. Lack of options available on the market

**Appendix 2**- Information included with survey questions

|  | **Carbon fibre** | **Metal implants** |
| --- | --- | --- |
| Composition | Carbon fibre-reinforced polyetheretherketone | Stainless steel, titanium and its alloys |
| Applications in Orthopaedics | Relatively new | Old and established |
| Acceptance by body | 100% fully biocompatible with no toxic, inflammatory or allergy possibilities | There is a small possibility of metal allergy. |
| Easier for the surgeon to evaluate x-rays and healing, both during surgery and after surgery? | Yes, as they are radiolucent | Sometimes is harder (obstructing visualisation of the nearby joint of the affected extremity) |
| Can be modified to better match the bone’s properties and potentially enhance healing? | Yes | No |
| Removal | Easier | Sometimes harder |
| Prone to breakage | Resistance +++ | Resistance ++++ |
| Cost | Higher | Lower |
